# Hyperinsulinemia: a Cause of Obesity?

**DOI:** 10.1007/s13679-017-0261-z

**Published:** 2017-05-02

**Authors:** Karel A. Erion, Barbara E. Corkey

**Affiliations:** 1 Obesity Research Center, Department of Medicine, Boston University School of Medicine, 650 Albany St, Boston, MA 02118 USA; 2 Division of Endocrinology, Department of Medicine, David Geffen School of Medicine, University of California Los Angeles, Los Angeles, CA USA

**Keywords:** Hyperinsulinemia, Insulin resistance, Hyperlipidemia, Energy efficiency, ROS, Redox

## Abstract

**Purpose of Review:**

This perspective is motivated by the need to question dogma that does not work: that the problem is insulin resistance (IR). We highlight the need to investigate potential environmental obesogens and toxins.

**Recent Findings:**

The prequel to severe metabolic disease includes three interacting components that are abnormal: (a) IR, (b) elevated lipids and (c) elevated basal insulin (HI). HI is more common than IR and is a significant independent predictor of diabetes.

**Summary:**

We hypothesize that (1) the initiating defect is HI that increases nutrient consumption and hyperlipidemia (HL); (2) the cause of HI may include food additives, environmental obesogens or toxins that have entered our food supply since 1980; and (3) HI is sustained by HL derived from increased adipose mass and leads to IR. We suggest that HI and HL are early indicators of metabolic dysfunction and treating and reversing these abnormalities may prevent the development of more serious metabolic disease.

## Introduction: Research has Failed to Explain Obesity

Current guidelines attribute obesity to overeating and inactivity based on the thermodynamic principle that change in mass = (input – output). Implementation of the NIH health guidelines from 1980: “avoid too much fat, saturated fat and cholesterol; eat foods with adequate starch and fiber”…coincided with a sharp rise in obesity. Unfortunately, the recommended therapy of dieting and exercise has not led to any amelioration of the high incidence of obesity.

Inadequacy of our conceptual understanding of obesity is documented by randomized clinical trial data showing the following:

- Overeating causes short-term weight gain but is often not sustained [1, 2••].
- Dieting leads to weight loss but is rarely sustained [1, 2••].
- Inactivity does not cause obesity.
- Exercise improves health but does not cure obesity [3••].

Some interesting observations indicate that there are differences among people who successfully defend their weight compared with those that gain weight more easily. Further evaluation of these extremes may lead to a greater understanding of obesity. We would suggest that such evaluations include the hormone and hormone response profiles, particularly to insulin.

## Obesity is Accompanied by Hyperinsulinemia, Hyperlipidemia and Insulin Resistance and is Often Presumed to Cause All Three but Could This be Incorrect?

Prior to the development of a severe metabolic disease, three interacting components are abnormal: (a) lipids are elevated, (b) basal or fasting insulin is elevated (HI) and (c) IR is present [4–6]. HI is more common than IR [7] and is a significant independent predictor of type 2 diabetes [8].

Insulin serves as the principal anabolic hormone responsible for proper storage of nutrients following ingestion of a meal. Many years of research have documented a positive relationship between obesity status and insulin levels in animal models and humans. Current dogma stipulates that elevated fasting and postprandial insulin levels serve to maintain proper glucose homeostasis in the face of systemic IR. Due to the glucocentric nature of research encompassing insulin secretion and signaling pervasive within the field, a coordinated effort has been made to enhance both effects [9]. The recent emergence of the concept of selective insulin resistance, in which tissues become resistant to insulin’s effect on glucose transport but remain sensitive to its lipogenic effect, has reinvigorated the hypothesis that HI may be a primary cause of weight gain that leads to obesity and type 2 diabetes [10]. Indeed, IR may be compensatory in the body’s response to prevent the metabolic syndrome. This section will examine the viability of the hypothesis that HI plays a primary role in the etiology of obesity based on cellular, clinical and epidemiological evidence.

## Body Weight is Maintained in the Short Term Despite Variation in Intake and Activity

The regulation of body weight involves many factors of varying degrees of importance but nevertheless appears to be stable in the short term despite dramatic variations in daily caloric intake and energy expenditure [11]. In the long term, body weight in humans follows an upward trajectory that averages 1–2 lb/year between the ages of 20 and 60 [12]. However, these averages do not explain the increasing incidence of extreme obesity and obesity in children or the fact that a minority of individuals maintain a stable weight throughout their life span. Differences have been noted among individuals during over- and underfeeding such that individuals who exhibit the greatest increase in energy expenditure during overfeeding are most resistant to weight gain [1] whereas those that decrease energy expenditure most during deprivation are most likely to gain weight [2••]. Thus, factors that regulate the ability to adapt in a way that maintains the weight trajectory may determine susceptibility to obesity. The role of insulin or HI in these responses is unknown. In particular, the sequence of changes in response to excess nutrients and during active weight gain have not been determined, nor have the differences among signals generated when overeating does not cause sustained weight gain.

## Alternative Testable Hypotheses Relevant to HI

### KO Animal Models

Rodents have two insulin genes: Ins1, the expression of which is mostly restricted to the pancreas, and Ins2, which displays expression in both the pancreas and the brain. Complete knockout of either gene does not alter circulating insulin nor impart any metabolic phenotype, likely due to compensation by the other gene [13]. Using mice completely lacking Ins2 and heterozygous for Ins1, researchers showed that these mice do not become hyperinsulinemic or obese on a high-fat diet [14]. Additionally, britening of white adipose depots was observed in mice with genetically reduced HI. Interestingly, suppression of HI via this genetic manipulation was found to provide life-long protection against obesity despite the eventual manifestation of an equivalent degree of HI [15]. These data imply that suppression of HI could provide protection against obesity later in life. Genetic prevention of HI also greatly blunts weight gain and adiposity in leptin-deficient *ob/ob* mice [16]. These data support the notion that prevention of initial weight gain by reduction of HI may be favorable to reduction of HI as a treatment for obesity. This topic has just been reviewed by the group of Johnson [17••].

A secondary approach to determining the role of HI in the manifestation of obesity is to inhibit insulin signaling. Use of the LoxP/Cre system allowed for the characterization of reduction of insulin signaling in specific tissues [18]. Several interesting and unexpected findings were observed [19]. Knockout of the insulin receptor in adipose tissue results in a severe reduction in fat pad mass and whole body triglyceride content [20]. Additionally, these mice are resistant to weight gain following ventromedial hypothalamus (VMH) lesion or as the result of normal aging and do not develop glucose intolerance even on a high-fat diet [20]. Wild-type mice on a high-fat diet have increased level of basal insulin signaling in peripheral tissues as assessed by Akt phosphorylation status [21]. Increased insulin signaling is a result of HI and is imperative for the accumulation of lipid within insulin-sensitive tissues. This concept is not restricted to peripheral tissues. Intracerebroventricular insulin administration increases fat mass and fat cell size, indicating that central insulin signaling can regulate peripheral lipid metabolism [22]. Increased insulin signaling in steroidogenic factor 1-expressing neurons of the VMH during obesity has been shown to regulate adiposity in mice on a high-fat diet [23]. In contrast to neurons of the arcuate nucleus, which become insulin resistant on a high-fat diet, those of the VMH remain sensitive to insulin thus allowing HI to drive peripheral lipid accumulation [24]. Lipid accumulation can be prevented through use of an inhibitor of phosphoinositide 3-kinase (PI3K), a kinase downstream of the insulin receptor [25]. Inhibition of PI3K also prevents the manifestation of IR within these tissues, supporting the hypothesis that lipid metabolites play an integral role in the manifestation of IR in obesity and is secondary to increased insulin signaling and HI. Importantly, inhibition of PI3K has been shown to reduce adiposity while sparing lean body mass [26]. The reduction in body weight and adiposity during treatment with inhibitors of PI3K is not due to a reduction in food intake but rather is due to increased energy expenditure, in part due to browning of white adipose tissue. These results in mice have also been translated to rhesus monkeys [26]. Daily administration of a PI3K inhibitor reduced adiposity and improved levels of glucose in serum in the absence of any detectable toxicity.

Alternative hypotheses involving specific proteins can be tested in animals using modern molecular and pharmacological techniques. It is critical in these studies to identify only physiologically relevant targets by using heterozygotes that exhibit a phenotype since homozygous phenotypes are analogous to rare monogeneic defects.

### Energy Efficiency, ROS and Redox Response to Altered Nutrient Supply

Modifying genes in many metabolically sensitive tissues can induce obesity and metabolic disease. Although IR has been assumed to reflect the key relevant pathosis [27, 28], evidence exists to also implicate signal transduction pathways of other tissues including pancreatic islets [29], liver [30], adipose tissue, brain, gut, vasculature, and muscle [31] that are sensitive to HI. Evidence supports an important role for each in metabolic homeostasis and thus a potential causative role in obesity. The possibility should be considered that pathosis results from contributions of many relevant tissues via a circulating redox communication system that coordinates responses and reflects shared control and regulation [32]. Such a master metabolic regulatory system would impact all organs in communication with the bloodstream.

- Pancreatic ß-cells to regulate insulin secretion.
- Adipose tissue to control lipid synthesis/breakdown, release of fatty acid and secretion of adipokines [33].
- Liver for gluconeogenesis and ketogenesis as well as lipid packaging and secretion [34–36].
- Gut and brain to control and integrate food consumption and satiety [37].

Elegant studies by Dean Jones and colleagues [38] and recent studies by our group [39••, 40•] are consistent with a role for circulating redox regulation of tissue-specific metabolism. However, it is not known how physiological HI impacts circulating redox since both anti- and pro-oxidant effects have been reported at different insulin concentrations [41]. Additional studies are also needed to differentiate cause from consequence. The possibility has not been tested that imposing a redox change, in vivo, will alter metabolism.

### Insulin Inhibits FA Oxidation and Lipolysis

A major function of insulin is inhibition of lipolysis, an appropriate response to food ingestion and the need to promote fat storage. Although increased oxidation of fat might be beneficial in obesity, the opposite is observed. Free fatty acid release from adipose depots (per gram of fat tissue) decreases in obesity [42]. In response to overeating, glucose is preferentially burned and fat is stored [11, 43]. Interestingly, one of the important changes induced by bariatric surgery is an increase in fatty acid oxidation [33, 34]. The potential benefit of stimulating fat oxidation through wasteful cycling or induction of rate-limiting enzymes of fat oxidation needs to be tested.

### Effects of HI on Neural Pathways

Insulin signaling is critical in both central and peripheral mechanisms of nutrient handling [35]. This concept is supported by the observation that overnutrition and obesity induce IR in specific brain regions [36]. In addition, increased fatty acid uptake has been documented in patients with metabolic syndrome that correlates with BMI and HI and reverses with weight loss [37] and bariatric surgery [44•]. Although the detailed molecular mechanisms and feedback circuitry are not fully established, it appears likely that neural pathways are major contributors to the adverse effects of HI. Future studies promise to provide greater detail on the specific roles of specific neurons and determine whether neural systems actually control body weight or rather serve as integrators of many signals.

## How Does HI Cause Obesity?

### Insulin is a potent storage signal to fat, brain, liver and muscle: Evidence for a causative role for HI in animal model obesity

The discovery of insulin by Best et al. in 1921 led to life-saving treatments for diabetics [45]. Early research demonstrated that injection of insulin into fat pads caused a dramatic expansion in tissue volume due largely to an increase in lipid storage [46]. It was quickly recognized that administration of insulin in rodents leads to expansion of total fat mass [47]. It was determined that this model of obesity resulted mainly from increased consumption of calories [48, 49]. However, enlargement of fat pads directly at the site of injection seemed to imply the possibility of a direct effect of insulin on lipid accumulation [50]. A second model of obesity popularized during this period was disruption of the VMH by chemical or electrolytic lesion. It was observed that this procedure induces HI and hyperphagia and quickly leads to obesity and increased fat mass. The HI and obesity observed following this procedure were initially believed to result solely from hyperphagia [51]. However, this belief was subsequently contradicted by multiple findings. First, destruction of ß-cells with the drug streptozotocin reverses hyperphagia and weight gain following the VMH lesion [52]. Exogenous administration of insulin recovers both the hyperphagia and weight gain, suggesting that HI is required for the manifestation of obesity following the VMH lesion. Second, prevention of hyperphagia following the VMH lesion is achieved by limiting caloric intake to that of sham-operated controls. This does not prevent the induction of HI or accumulation of fat [53, 54]. Lastly, VMH lesions in weanling rats do not induce hyperphagia but still result in HI and accumulation of fat [55]. Multiple groups noted a direct correlation between the change in body weight and the degree of HI following VMH lesion [56, 57]. Pharmacological reduction of insulin alleviates the hyperphagia and weight gain in multiple species [58, 59]. Indeed, hypothalamic-related obesity induced by VMH injury in children undergoing cancer therapy can be partially alleviated by reducing insulin levels [60, 61].

Genetic and diet-induced models of obesity have largely replaced the VMH lesioned model. The most common genetic models of obesity are the *ob/ob* mouse and Zucker fatty rat, both of which harbor mutations in the leptin or leptin receptor gene rendering the hormone nonfunctional. These animals display extreme HI, hyperphagia and obesity. At least part of the HI in this model can be attributed to the fact that leptin normally acts to suppress insulin synthesis and secretion in ß-cells [62, 63]. Enhanced lipogenesis has been observed in these animals as early as 15 days after birth and is accompanied by HI [64]. The HI that arises in *ob/ob* mice precedes insulin resistance and substantial weight gain [65]. Replacement of leptin in these animals completely normalizes hyperphagia, HI and abnormal adiposity [66]. However, this is not simply due to a normalization of caloric intake. Pair-fed *ob/ob* mice eating a normal caloric intake still display extreme HI and increased adiposity compared to control mice [67]. Several drugs have been used to directly assess the role of HI in the obesity of rodents with mutations in leptin. Diazoxide, a K_ATP_-channel agonist that inhibits membrane depolarization, reduces nutrient-induced insulin secretion from ß-cells. Supplementation with diazoxide reduces adiposity and causes weight loss while simultaneously improving lipid profiles [68]. Despite the reduction in insulin secretion, these rodents have reduced glycemia and improved glucose tolerance [69]. Diazoxide enhances both insulin sensitivity and energy expenditure. As insulin levels decline, fatty acid oxidation increases thus relieving the inhibition of insulin signaling known to occur by certain fatty acid metabolites [70]. Thus, in this model, it appears that HI precedes insulin resistance and may in fact contribute to it by directly downregulating the insulin receptor or through inhibiting insulin signaling by increasing lipid accumulation.

### Documentation of HI in Humans

Data supporting the HI-induced obesity hypothesis is less available in humans. However, certain racial ethnicities known to have very high levels of circulating insulin are also known to be at increased risk for the development of obesity. It was first recognized in the 1950s that Pima Indians, a tribe in the American Southwest, display abnormally high rates of obesity and diabetes [71]. It was subsequently determined that this population displays abnormally high insulin response to nutrients inducing HI [72]. Pima Indian children have significantly higher fasting insulin levels, which is predictive of the risk for becoming obese [73, 74]. The ability of HI to predict obesity has been repeated in ethnicities other than Pima Indians, including a recent study in Chinese children [75, 76]. African American children, an ethnicity with a particularly high rate of obesity, are hyperinsulinemic compared to Caucasian children [77, 78]. It is important to note that this finding is less consistently observed in adult populations, suggesting a key role for insulin in determining weight gain in children but less so in adults [76, 79, 80].

## Effect of Reducing HI

### Both pharmacological and nutritional approaches have been used to reduce hypersecretion of insulin as a method for weight loss

Diazoxide promoted enhanced weight loss in obese adults when combined with an 8-week energy-restricted diet [81]. Compared to patients on placebo, those receiving diazoxide lost more fat and maintained a higher fat-free:lean mass ratio. Although there was no improvement in glucose tolerance observed compared to that in placebo, there were also no adverse effects despite a large reduction in postprandial insulin secretion. In contrast, a second trial with a similar design did not observe any significant additional weight loss with diazoxide supplementation [82]. The basis for the discrepancies between these two trials is currently unclear. However, a much more profound effect of diazoxide on postprandial insulin secretion was observed in the trial in which diazoxide induced weight loss. A trial testing the ability of 6-month administration of a somatostatin mimetic to induce weight loss in the obese noted a positive correlation between dose and effectiveness [83].

Preventing or reversing HI via nutritional intervention as a means to treat obesity has garnered interest in recent years. The concept of glycemic index, a measure of rate of carbohydrate absorption, has been a popular area of research in the field of nutrition [84]. Following consumption of foods with a high glycemic index (sugars), the resulting spike in glucose leads to exaggerated insulin secretion and relative HI, as glucose is the main secretagogue for insulin secretion. Consumption of a diet high in simple carbohydrates has been shown to consistently increase adiposity in rodents [85, 86]. These results have proven challenging to replicate in humans and have resulted in variable results and conclusions as to the ability of glycemic index to modify body weight and adiposity [87•]. It has been proposed that patients with high degrees of relative HI may benefit the most from a low-glycemic index diet [88]. Indeed, people exhibiting HI, after weight loss with a hypocaloric diet, were most at risk for weight regain [89]. *Despite this ambiguity*, *recommendations to reduce dietary simple carbohydrates (sugars) make logical sense because they serve no essential nutritional need.*

**Fig. 1:**
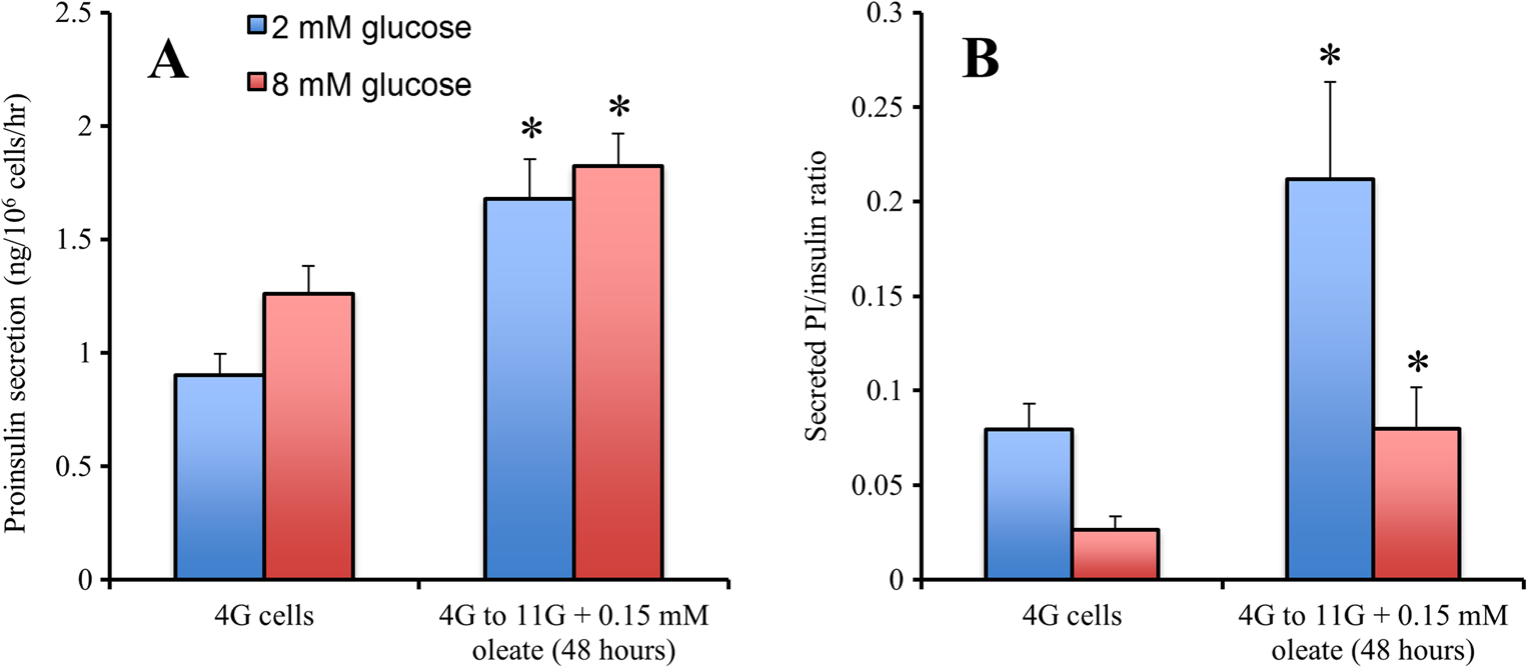
Chronic exposure to excess glucose and oleate increases proinsulin secretion. **a** INS-1 cells cultured in 4 mM glucose have lower proinsulin secretion at both basal (*2 mM*) and stimulatory (*8 mM*) glucose compared to those cultured at 11 mM glucose and 0.15 mM oleate for 48 h. **b** 4G cells have a lower ratio of secreted PI/insulin ratio compared to cells cultured at 11 mM glucose and 0.15 mM oleate (*n* = 6 independent experiments). Data are mean ± SEM. *p < 0.05 versus control (4G cells). Data are mean ± SEM. *p < 0.05 versus respective control (Student’s *t* test)

HI may play a causative role not only in the development of obesity but also in the ß-cell dysfunction that precedes type 2 diabetes. Obesity leads to HL that further exacerbates the HI via the reduction of hepatic insulin clearance [90]. We have previously shown that the hypersecretion of insulin following chronic exposure to elevated fatty acids impairs the ability of the ß-cell to adequately respond to acute nutrient stimulation [91••]. ß-cells secreting a high percentage of maximal capacity at basal glucose exhibit reduced glucose-stimulated insulin secretion [34]. Additionally, HI is accompanied by altered insulin processing. Figure 1 demonstrates that culture of clonal ß-cells, under conditions that increased cellular lipid and induced HI, also increased proinsulin (PI) secretion and exhibited an elevated PI:insulin ratio. Increased PI secretion may be due to impaired processing of PI or reduced time for processing due to a high secretory rate. The ratio of circulating PI:insulin is also increased in obesity and is predictive of the development of IR and type 2 diabetes [92–94]. Interestingly, induction of overnight ß-cell rest with somatostatin in type 2 diabetic patients normalized the increased PI:insulin ratio [95].

### Effect of Bariatric Surgery on HI

*Bariatric surgery is currently the only way to reliably induce sustained weight loss* in obese humans. Several different types of bariatric surgery are currently being used including Roux-en-Y gastric bypass, vertical sleeve gastrectomy and biliopancreatic diversion. All of these procedures result in substantial weight loss and improvement of the metabolic syndrome and type 2 diabetes. The weight loss induced by bariatric surgery is not solely due to the restrictive nature of the procedure. Recent evidence suggests that the body weight “set point” defended is changed following bariatric surgery [96]. Bariatric surgery induces a number of seemingly diverse physiological changes from gene expression to the palatability of certain foods to the microbiome [97–99]. Hormonal changes also play an integral role in metabolic changes resulting from the surgery. The majority of research has focused on changes in glucagon-like peptide one and satiety hormones. However, another hormonal change that occurs rapidly following surgery is a sharp drop in steady-state insulin. Resolution of hyperinsulinemia is one of the earliest events following bariatric surgery and precedes the recovery of peripheral insulin sensitivity [100]. Directly testing the role of insulin reduction in the resolution of obesity and type 2 diabetes following bariatric surgery will need to be addressed in animal models.

## Conclusion

Our goal in this brief perspective has been to question the dogma explaining obesity and underpinning the therapeutic guidelines for treatment that are largely unsuccessful. We offer an alternative hypothesis that requires further testing and further suggests that if indeed our hypothesis is correct, early treatment of the HI and HL that precede metabolic dysfunction may successfully treat obesity and prevent complications. Since the dramatic increase in obesity has occurred in the last 50 years, we suggest that a focus should include new elements in our environment that may serve as potential obesogens through effects on any of the communicating metabolically sensitive organs: the brain, liver, adipose tissue, islets of Langerhans, gut and cardiovascular system.

## Abbreviations

HI: Hyperinsulinemia
HL: Hyperlipidemia
IR: Insulin resistance
PI3K: Phosphoinositide 3-kinase
VMH: Ventromedial hypothalamus
PI: Proinsulin
